# The Mitigating Toxic Stress study design: approaches to developmental evaluation of pediatric health care innovations addressing social determinants of health and toxic stress

**DOI:** 10.1186/s12913-021-06057-4

**Published:** 2021-01-19

**Authors:** Julie S. McCrae, Jo Ann L. Robinson, Angeline K. Spain, Kaela Byers, Jennifer L. Axelrod

**Affiliations:** 1 Chapin Hall at the University of Chicago, 1313 E. 16th Street, Chicago, IL 60637 USA; 2 University of Connecticut, 348 Mansfield Road U1058, Storrs, CT 06269-1058 USA; 3 University of Kansas, 1545 Lilac Lane, Lawrence, KS 66045 USA; 4 The Chicago Community Trust, 225 N, Michigan Avenue, Suite 2100, Chicago, IL 60601 USA

**Keywords:** Toxic stress, Social determinants of health, Infants, Pediatric primary care, Early childhood

## Abstract

**Background:**

Health care administrators must establish and promote effective partnerships with community agencies to address social determinants of health, including reducing exposure of infants and young children to chronic stress. Because infants’ experiences are inextricably tied to their caregivers, an important target for mitigating “toxic” stress exposure in early childhood is through reducing parents’ experiences of chronic stress in addition to protecting children from direct experiences of harm such as physical or sexual abuse. Conducting screening to identify when children are exposed to early life adversity is a first step; connecting families to needed support services is an essential component to addressing identified challenges. This paper presents the methodology of a three-year study of health care systems innovations designed to engage and support parents of infants to prevent and mitigate children’s toxic stress exposures.

**Methods:**

Key study features included: 1) multi-component study in five U.S. communities and nine pediatric health care clinics and the families they serve, 2) a developmental evaluation approach to describe how innovations are experienced over time at three levels—community systems, pediatric providers, and families, and 3) rapid cycle feedback conducted with communities, clinics and families to co-interpret data and findings. Data sources included: 1) focus groups and interviews with community stakeholders, clinic staff, and families, 2) electronic health record and Medicaid services data extracted to assess health care quality, utilization, and financial impact, and 3) clinic-recruitment of 908 parents of infants in a longitudinal survey. Results. The sample is briefly characterized based on responses to the enrollment phase of the parent survey.

**Conclusions:**

We discuss the study design elements’ contribution to generating evidence needed by innovators, communities, and clinics to modify and sustain investments in these innovations to prevent or mitigate the effects of exposure to toxic stress on young children.

## Background

Health care screening and referring families to resources are critical roles for pediatric health care practices to consider as part of addressing social determinants of health (SDoH). Social determinants of health are the conditions or circumstances in which people are born, grow, live, work, and age, and the wider set of forces and systems shaping the conditions of daily life [1, 2]. This includes social, cultural, economic, and political forces that contribute to health disparities [3]. During the first post-natal year, these social determinants may help or hinder families’ access to services and could play a significant part in parents’ experience of strain [4, 5]. Infants can experience much of the same social stress and its impact as their parents. Poverty, inadequate food or nutrition, or exposure to violence, for example, can prompt a physiological stress response, primarily indicated in the amount of cortisol that is produced and for how long [6]. Infants may also be impacted by chronic stress through the relationship with their parents; developmentally supportive parenting can help buffer children from stress by promoting children’s early development [7]. The term “toxic stress” is used to refer to the strong, frequent, or prolonged activation of the body’s stress response systems in infants while not being buffered by protection from a supportive adult [8]. Protective factors including supportive relationships, and access to basic needs such as high-quality food and transportation, can mitigate chronic stressors and promote resilience. These positive childhood experiences in the face of adversity have been shown to independently predict adult health [9]. Assessing parent experiences of stress and burden can ensure timely access to services and promote child health. The American Academy of Pediatrics and the American Academy of Family Physicians are just two of the professional practice organizations in the U.S. that underscore pediatric and family health care providers’ role in addressing the social needs of families [10, 11].

Because the origins of stress are multi-faceted and multi-determined, reducing the development of toxic stress through enhancing protective factors must also be multi-systemic. One challenge facing pediatric health care is the effective integration of screening for families’ social needs, referring to services (such as food banks or housing) and facilitating family access to services. Child health practitioners and managers frequently endorse recommendations for screening, however, they also report that community services (e.g. early childhood behavioral health) are often limited or unavailable and therefore are reluctant to implement screening guidelines [12, 13]. Health care providers also do not typically have the capacity, nor the financial model including staff roles and time necessary to monitor the available service array in their community. The impact of innovations in health care to prevent or mitigate toxic stress in children is therefore influenced by the available service continuum and effective partnership across health care and community-based services.

Latino families are even more disenfranchised from health care and therefore more likely to have less access to referral and formal support networks. Child health insurance programs have extended well-child and illness care to millions of uninsured families throughout the U.S. [14], making more apparent the many unaddressed physical health and social needs they face. Latino families in the U.S. have the highest uninsured rates, compared to other racial or ethnic groups, with only 49% reporting insurance coverage in 2017 [15]. As a marker of socioeconomic adversity, lack of health insurance often co-occurs with other social needs including insecure housing and food [16, 17]. There is evidence that accessing health and economic supports decreased from 2015 to 2018 among Latino families [18]. Public charge rules that were adopted in 2019 also created a chilling effect that further reduced Latino families’ enrollment in benefits.

In this paper, we describe a study that triangulates three important perspectives (community systems, health care clinics, and families) on strategies for promoting child health through bolstered partnerships between health care and early childhood systems to support families of infants. Participating health clinics serve predominantly Latino families. The study is designed to examine two approaches to pediatric health innovations that are being implemented in the U.S. to prevent and mitigate conditions related to exposure to early childhood adversity and the lack of protective factors. This study documents systems change, healthcare provider behavior, and family engagement with health and community services in relation to health and stress outcomes over time. Our theory of change asserts that the effects of these innovations are first at the systems level, influencing collaboration, provider behavior and family engagement efforts. Strengthening these systems thus provides families with increased supports for both concrete and health needs. This in turn increases parent sense of agency, prompting behavioral changes that promote child health and well-being.

Developmental Understanding and Legal Collaboration for Everyone (DULCE; 19) and Improving Screening, Connections with Families, and Referral Networks (I-SCRN; 20) are two approaches designed to increase the pediatric care provider’s role in strengthening family supports by identifying family needs in areas of SDoH and referring and linking families to resources. Both innovations involve: screening during health care visits; a multidisciplinary team; enhanced conversations with families about child development, maternal depression, and SDoH; and collaboration with a local system of care. DULCE was developed, implemented and tested in a randomized control trial of 330 parents of infants in Boston, showing that the intervention accelerated family access to concrete resources and that participants were more likely to complete their 6-month immunizations by age 8 months and less likely to have visited the emergency department by age 6 months [19]. DULCE embeds a family specialist to work in tandem with pediatricians and families during the first 6 months of preventive health care visits, and also uses a legal partnership approach. The legal partners provide: training and support for the family specialist around screening for legal issues associated with social determinants of health, legal information, and referrals for families for legal intake if appropriate. Attorneys also participate in a weekly care planning meeting for families and can provide immediate legal advice and represent families in court. DULCE was implemented in seven of the nine clinics in our study.

The American Academy of Pediatrics’ (AAP) I-SCRN is a quality improvement collaborative to support pediatric primary care practices in the implementation of effective processes for screening, discussion, referral, and follow-up of child development, autism spectrum disorder (ASD), maternal depression, and social determinants of health [20, 21]. I-SCRN was implemented in two of the nine study clinics. Practices participated in I-SCRN over 1 year with other collaborative members and the AAP, involving virtual and in-person learning opportunities, in-clinic practice teams centered on early childhood screening, and a continuous quality improvement process based on monthly chart reviews and surveys [21]. Using the monthly chart reviews (10 per month per clinic for well-child visits at 9, 18, and 30 months) among 19 participating practices, significant increases in screenings conducted were observed, as well as the proportion of visits where child development, ASD, maternal depression, and SDoH were discussed, and referrals for developmental and maternal depression services were made [21].

The two clinic innovations by design promote access to services that are difficult for the most stressed, marginalized populations to access while beneficial for women and children across socio-economic circumstances. Clinics can both refer families directly to services and leverage centralized referral systems like Help Me Grow (HMG) that build on existing community resources to develop a comprehensive network of early childhood services in a community [22]. The HMG model includes a centralized access point (e.g. call center), family and community outreach, child health care provider outreach, and referral tracking. Help Me Grow was available to four of the five study communities.

### Study overview

This paper describes a developmental evaluation study using mixed methods to understand the experiences of five communities, nine health care clinics, and 908 families in the implementation of two healthcare/early childhood systems innovations to promote resilience and reduce stress in the child’s family during the first year of life. The study’s evaluation strategy is rooted in prevention science and incorporates two approaches critical for evaluating innovation. The first approach is an articulation of the multiple levels at which an innovation operates, including documenting the variance between the intended versus actual implementation experienced by stakeholders. Second, because outcomes are rarely driven by single causes, it is important to document the paths of theoretically meaningful mediators of outcomes [23]. This evaluation plan developed for the study highlights multiple levels (community systems, clinics, and families) and pathways at which the innovations operate through the project design, measurement, and analysis strategy. We capture change over the time period of the study using a developmental evaluation approach [24, 25]. Focus groups and interviews (qualitative methodology) with parents, clinic staff, and early childhood community leaders complemented parent longitudinal survey interviews and electronic health records extracted for the study.

**Fig. 1:**
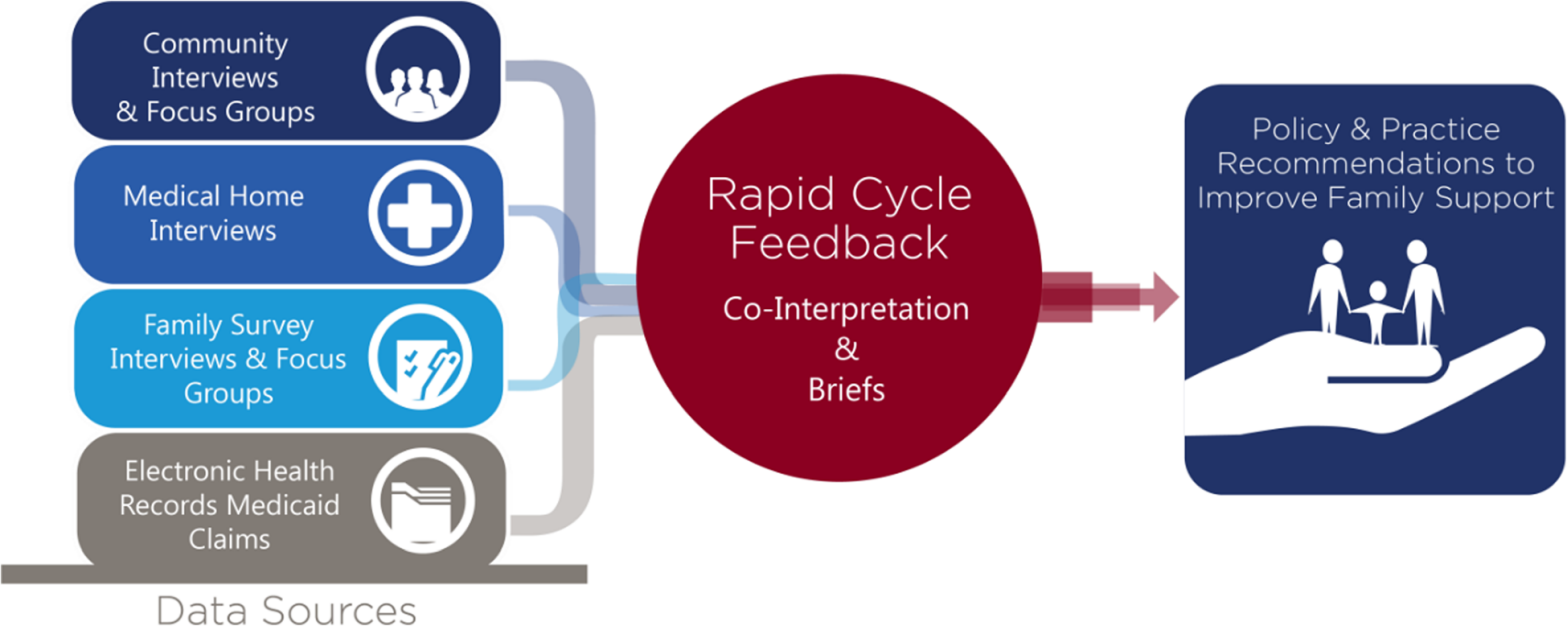
Developmental Evaluation Approach

Figure 1 illustrates the evaluation’s focus on rapid-cycle feedback and co-interpretation approaches that are part of the developmental evaluation approach. This model, adapted from a synthesized member checking methodology for qualitative research, captures evolution of the health innovations and the communities in which they are embedded [26].

## Methods

The five communities in the study were chosen from a larger set of communities engaging in an early childhood systems transformation effort in 2018, The Early Childhood Learning and Innovation Network for Communities (EC-LINC; https://cssp.org/our-work/project/early-childhood-learning-and-innovation-network-for-communities/) led by the Center for the Study of Social Policy (CSSP). Participating communities in EC-LINC bring together early childhood brokering or hub organization with specific community partners to strengthen systems and services to better meet the needs of families with young children. In 2018, there were 10 EC-LINC communities. Those in the study were chosen because of their interest in strengthening how they partnered with health care systems to promote family protective factors. During this time, pediatric health care clinics within each community also self-selected and received resources to implement DULCE or I-SCRN as part of program developers’ larger efforts to implement the models. Clinics that opted in to DULCE or I-SCRN in the communities were then approached to participate in the study. DULCE clinics were recruited by the research team and CSSP, and interested I-SCRN clinics responded to a recruitment flyer distributed by the research team and the AAP. The final sample included nine clinics—seven implementing DULCE and two implementing I-SCRN. All study procedures were approved by the University of Chicago Social Services Administration-Chapin Hall Institutional Review Board (IRB).

We describe each data collection method next, beginning with qualitative data collection methods across various stakeholder groups over time and then transitioning to quantitative methods including electronic health record extraction and longitudinal survey methods. For the quantitative data collection with families, community-based Field Interviewers completed the interviews (see Family Longitudinal Interviews section). For qualitative interviews and focus groups, data collection was conducted by a research team led by the PhD-level qualitative study lead. Interviews and focus groups were conducted in English, Spanish and in one community Haitian-Creole, and lasted 45 to 90 min and were audiotaped and transcribed. Table 1 describes the levels of primary data collected, data sources, methods, and number of participants per time point. Data were collected in each of 3 years (2018–2020) of the study. In 2018, interviews and focus groups were conducted with national leadership of the three innovations, EC-LINC leadership in the five communities, and leaders of the clinic innovation teams. We commenced family longitudinal interviews in March 2018 completing the final longitudinal interviews with families in February 2020. In 2019, we focused data collection at the community and clinic levels.

**Table 1:** Data collected at the national, community system, pediatric healthcare clinic, and family levels

Levels and Data Sources	Sample Description	Method^**f**^	No. of participants by time point^**g**^		
			T1	T2	T3
			*2018 2019 2020*^*g*^		
*National Innovations*	National leadership of DULCE, I-SCRN and HMG innovations	I	16		
*Community Systems*					
Early Childhood Lead Organizations	EC-LINC Leadership in each community	I	11		
Community Service Providers	Mental health, child care/early education, home visiting, and public health providers^a^	FG		39	
Families	Parents or caregivers with early childhood service experiences (e.g. home visiting, early intervention)^a^	FG		58	
Centralized Referral Study	A sample of three community Help Me Grow (HMG affiliates^b^				
Help Me Grow Staff	Leadership staff within each HMG affiliate	FG		13	
Community Service Providers	Mental health, child care/early education, home visiting, or public health services providers that referred families to HMG^b^	FG		24	
Healthcare Providers	Pediatric providers that referred families to HMG	I		11	
Families	Parents/caregivers who had utilized HMG^b^ within the past six months	FG		17	
*Pediatric Healthcare Clinics*					
Innovation teams	DULCE and I-SCRN team members^c^	I	54		
Clinicians	Health care staff providing clinical services, regardless of being involved in the DULCE or I-SCRN innovations^d^	S		63	
Families	Parents/caregivers who received pediatric health care services at each clinic within the past year	FG		110	
*Families (n = 908)*	Parents of newborns age 2 to 24 weeks who received pediatric health care services at each clinic^e^	SI	888^h^ (98%)	764 (84%)	707 (78%)

^a^Community service providers were nominated for focus groups by the early childhood lead organization; families with experience accessing early childhood services were nominated for interviews by either the early childhood lead organization or community service providers

^b^HMG affiliates (*n* = 3) were selected based on geographic diversity (2 urban and 1 rural community) and years of HMG implementation in response to a request for applications. Community service providers, healthcare providers, and families with recent referral experiences with HMG were nominated by HMG staff

^c^Includes the physician or nurse practitioner innovation champion, DULCE family specialist (as applicable), DULCE legal partner (as applicable), DULCE early childhood lead (as applicable), social worker(s), clinic administrator, and in some clinics behavioral health leadership

^d^Includes physicians, pediatricians, nurse staff, medical assistants, and physician assistants

^e^908 families were recruited from the 9 participating health clinics by study field interviewers February 1, 2018-May 31, 2019

^f^*I* Interview, *FG* Focus group, *S* Survey, *SI* Survey Interview. Family focus groups were conducted in both English and Spanish (15 total English FGs and 13 total Spanish FGs)

^g^Time points refer to data collection as planned and may not precisely match calendar years of data completion

^h^For family survey interviews, T1, T2 and T3 are infant age 2 weeks-6 months (T1), 7–11 months (T2), and 12–15 months, with at least 30 days between interviews

### National Innovations

Interviews were completed with 16 of the DULCE, I-SCRN and HMG national teams members (*N* = 16). The interviews centered on three topics: (1) lessons learned about support clinic innovation; (2) the conceptual approach to addressing toxic stress and family engagement; and (3) emerging challenges and opportunities for the field. The purpose of this investigation was to understand the innovation model and implementation plans at the outset of the study in 2018.

### Community Systems

Multiple levels of community-level data were collected to understand each community’s early childhood service system. First, we conducted telephone interviews with 1–2 EC-LINC leadership staff per community (*n* = 11). EC-LINC leaders were asked about the vision for early childhood-healthcare partnerships and lessons learned from DULCE and I-SCRN implementation and from supporting Help Me Grow as a centralized referral network.

We then conducted focus groups with community service providers (*n* = 36) recruited by the early childhood organizations such as mental and behavioral health providers, child care and early education providers, home visiting providers, regional centers, and public health departments. The focus groups inquired about approaches to mitigating toxic stress, healthcare-early childhood partnerships, policies and practices related to screening and referral for social determinations of health, and family engagement strategies.

To understand family perspective, we asked the early childhood organizations and providers to nominate and invite parents or caregivers who could speak to experiences accessing early childhood services to a focus group. We conducted 8 focus groups (4 in English and 4 in Spanish) with a total of 58 parents/caregivers. Parents/caregivers had diverse experiences with the early childhood and healthcare systems (participation in home visiting, healthy pregnancy programs, or as community leaders). The 90-min focus groups inquired about experiences with community providers and pediatric primary care in their community.

Finally, we partnered with one participating state to understand the role of Help Me Grow in facilitating the referral network and bridging health care and community-based services for families. The purpose was to learn from HMG staff, community providers, health care providers, and families about their experiences with the roles of HMG as a centralized early childhood referral system. Three HMG affiliate locations responded to a request for applications and were selected for geographic representation and diversity in the number of years of HMG implementation. Within the three communities (two urban, one rural/suburban), we conducted 45-min telephone interviews with HMG staff and health care providers (*n* = 11). We conducted 3 English and 2 Spanish focus groups with families (*n* = 17), and 3 focus groups with community providers (*n* = 24). Focus groups were 60 min and asked about perceptions of facilitators and barriers to participating in the centralized referral system and critical opportunities to strengthen referral pathways in each community. Healthcare provider interviews also centered on these topics.

### Pediatric Health care clinics

We collected data from three groups at each clinic: (1) innovation team members (DULCE or I-SCRN), (2) clinicians, and (3) families. Innovation team members (*n* = 54) were interviewed in 2018 to gain perspective about early experiences with implementation. The interviews asked about core innovation components, opportunities, and barriers to integrating SDoH screening into the practice’s standard of care. Participants included the physician or nurse practitioner “champion,” trained family specialists, social workers, clinic administrators, and other team members supporting implementation. In 2019, we surveyed a broader group of clinic staff. We invited staff at each clinic (physicians, pediatricians, nurse staff, medical assistants and physician assistants) to complete an online survey, resulting in 63 completed surveys. The survey was designed to measure clinician attitudes and practices related to early childhood healthcare services, screening, and perceptions of occupational burnout (see Table 1 for the measures used).

Finally, we asked clinic innovation team members and early childhood organizations to nominate parents/caregivers to be invited to a 90-min focus group. Reflecting the languages preferred by families served by the clinics, we conducted 7 focus groups in English and 8 focus groups in Spanish with 110 total participants and at least one focus group conducted at each clinic. Focus groups asked about experiences with screening and referral for social needs in the clinic setting, and interactions with community providers following referrals.

### Family Longitudinal Interviews

Aiming to enroll 110 parents/caregivers of infants per clinic, we recruited 908 parents/caregivers to participate in three in-person survey interviews before their infant’s 2-year birthday (92% of recruitment goal). The first interview was conducted when infants were 2 weeks to 6 months of age, followed by midpoint and final interviews at infant age 7–11 months and 12–15 months, respectively. After the initial interview, each follow-up interview was scheduled to allow a period of no fewer than 30 days between interview waves.

Recruitment was facilitated through close collaboration with clinics, and conducted by 12 community-based field interviewers. Field interviewers were bicultural and fluent in English and Spanish and in one community: Haitian-Creole. Recruitment strategies were discussed, reviewed, and refined collaboratively with clinics throughout the study time period to accommodate clinic flow and patient protection. Early challenges in recruiting families led to highly effective collaborative decisions. Recruitment techniques included waiting room recruitment (field interviewers approached families in the waiting room), warm hand-offs from clinic staff to the field interviewer, clinic staff introducing the study to parents by phone followed by field interviewer phone contact, and texting parents. Field interviewers used study-issued surface notebooks for all communication and data collection with families.

Data sharing agreements enabled collaboration between researchers and clinic staff and between clinics and field interviewers. The study included extensive field interviewer supervision by a bilingual and bicultural researcher using real-time information and data about contact attempts and interview completion rates with parents to support field interviewers in reaching their recruitment and retention goals. Supervision also addressed the challenges experienced by Field Interviewers when engaging with caregivers and their infants to maintain boundaries. Field interviewers also were provided monthly summaries of their progress-towards-goals based on the targeted recruitment number, also discussed during monthly individual supervision. Study retention was 84% at midpoint, and 78% at the final interview (Table 1).

### Family longitudinal interview survey measures

Table 2 presents the measures set administered with parents/caregivers. Interviews included a group of established and newly developed measures of five areas of family experiences: (1) the home and neighborhood environment, (2) parent adverse childhood experiences, (3) resilience and personal mastery, (4) parenting, and (5) access to social support and resources. Measures were selected to provide a snapshot of comprehensive household and environmental risk and protective factors in infancy among families receiving pediatric care in participating clinics. The study will use this snapshot to examine how clinic engagement and support for impacted families entering pediatric care with diverse constellations of strengths and needs. Among the measures assessed at enrollment, we collected parents’ report of Adverse Childhood Experiences (ACEs), using the expanded scale developed by the Center for Youth Wellness [33]. Though primarily interested in current household and environmental circumstances, caregiver ACEs are associated with factors directly impacting the child’s environment, including caregiver mental health, substance use, and relational well-being and were therefore important to capture as part of the overall assessment of the household. Licenses were obtained as required for use of six study instruments (see Table 2).

**Table 2:** Family longitudinal and clinic staff survey measures

Measure	Construct	Description
**Family Measures**		
*Home and Neighborhood Environments*		
Safe Environment for Every Kid–Parent Questionnaire (SEEK™) [27]	Environmental Safety	14-item screening tool used by child healthcare professionals for identifying psychosocial risk factors. Validated among pediatric primary care populations [28–30].
Housing Quality Scale [31]	Perception of housing quality	5-item self-report scale of housing quality including physical condition of current housing. Reliability of this measure was established by developers; reliability confirmed and validity established through pilot testing in this study.
Housing Instability Index [32]	Housing stability	10-item self-report scale assessing instability over the previous 6 months, including items related to eviction, difficulty paying for housing, and transiency. Reliability of this measure was established by developers; reliability confirmed and validity established through pilot testing in this study.
Neighborhood Disorder Scale [31]	Neighborhood safety climate	5-item self-report assessing perceptions of neighborhood disorder (e.g. drug use, graffiti, loitering, and buildings in disrepair). Reliability of this measure was established by developers; reliability confirmed and validity established through pilot testing in this study.
Neighborhood Danger Scale [31]	Perception of neighborhood safety	5-item self-report scale measuring perception of safety at home at night, on neighborhood stress during the day, and on neighborhood stress at night. Reliability of this measure was established by developers; reliability confirmed and validity established through pilot testing in this study.
*Experiences of Life Stressors*		
Center for Youth Wellness–Adverse Childhood Experiences Youth Version (adapted) [33]	Caregiver experiences of childhood adversity	19-item self-report tool measuring childhood experience with stressful live events such as physical, emotional, and sexual abuse, physical and emotional neglect, household dysfunction, parental incarceration or death, and. Longitudinal studies are currently working to establish validity and reliability for this tool. Results reported in the current study are twelve of the nineteen total items that were asked of caregivers during the first interview.
Functional Impact of Life Events for Parents (FITS-P) [34]	Functional impact of exposure to toxic stress	4-item binary self-report scale assessing caregiver functional impact resulting from exposure to toxic stress, developed and validated for this study.
*Experiences of Resilience and Personal Mastery*		
Connor-Davidson Resilience Scale (CD-RISC) [35]	Resilience	25-item Likert self-report scale of resilience widely used across service systems and populations with established psychometric properties including test-retest and internal consistency reliability, and convergent, and divergent validity with a wide breadth of established measures.
Pearlin Mastery Scale [36]	Parental mastery	7-item Likert self-report scale assessing individual mastery defined as the extent to which individuals perceive their life as under their own control. This scale assesses beliefs about mastery over current circumstances, future possibilities, and ability to solve problems independently. This scale has established internal consistency and test-retest reliability, and correlations with other scales and variables [36–38].
*Parenting Experiences*		
Infant Development Questionnaire (IDQ) [39]	Infant development knowledge	15-item binary-response self-report measure of maternal knowledge of infant development. Psychometric properties of this measure have not been established.
Parental Stress Scale (PSS) [40]	Parenting stress	18-item Likert self-report measure of stress in parenting. Psychometric properties for this scale are robust, suggesting that the scale has sufficient internal consistency reliability, convergent validity with other parenting stress measures, and divergent and predictive validity.
Parenting Interactions with Children: Checklist of Observations Linked to Outcomes (PICCOLO) [7]	Development-ally supportive parenting behaviors	29-item brief observational measure of four positive parenting practices: affection, responsiveness, encouragement, and teaching. This tool has been shown to be reliable and valid for use with parents [41]
*Child Health and Development*		
Brief Infant-Toddler Social and Emotional Assessment (BITSEA) [42]	Child social-emotional development	42-item self-report screening tool for identifying child social-emotional and behavioral problems and delays among children ages 12 through 36 months of age. The BITSEA is a well-established tool with robust psychometric properties, including excellent test-retest reliability, concurrent and predictive and discriminant validity and acceptable specificity [43].
National Survey of Children’s Health (Adapted) [44]	Child health status	The NSCH is designed to produce national and state-level data on the physical and emotional health of children 0–17 years old in the United States. Number of items vary due to response but estimated average time for collection of data per survey is 2.5 h. Psychometric information is generally not available for the 14-month wave, but is for the 24- and 36- month waves.
*Access to Social Support and Resources*		
Health Families Parenting Inventory Mobilizing Resources Subscale [45]	Caregiver ability to access and use community resources	5-item Likert subscale of the 63-item self-report measuring assessing parenting domains. Assesses caregiver ability to access and use community and societal resources. The full scale has well-established psychometric properties, including construct validity, internal consistency reliability, and promising results related to sensitivity to change [46].
DULCE Social Connectedness [19]	Social connections in the community	2-item self-report scale assessing caregiver access to emergency care and support when needed. Psychometric properties of this scale have not been tested [19]..
Early Head Start Community-Based Partner Measure [47]	Use/access to community-based services	A subset of items adapted from the 36-month exit interview conducted by Mathematica Policy Research, Inc. and the U.S. DHHS Administration on Children, Youth, and Families as part of the 2001 Early Head Start Evaluation. While not psychometrically validated, this measure provides an established means of inquiring about connection to a variety of community-based family support programs.
Help Me Grow Protective Factors Survey (adapted) [47]	Protective factors	10-item self-report measure of caregiver knowledge of child needs, perceived access to support, and functional and relational coping. We pilot tested this measure for this study and established construct validity and internal consistency reliability (α = .93).
*Patient Engagement with Clinic*		
Clinic Inclusivity and Regard for Parents Scale (CIRP)	Inclusion in primary care decisions	11-item self-report Likert scale assessing parenting perceptions of engagement and inclusion in healthcare decision-making for their children. The CIRP was developed and pilot tested for this study. We established construct validity as well as internal consistency reliability (α = .93).
Parent Patient Activation Measure (PPAM) [48]	Caregiver activation in pediatric primary care	13-item self-report Likert scale that measures healthcare activation – or ability to manage a child’s health and healthcare. The PPAM was developed based on the Patient Activation Measure [49], which has well-established psychometric properties of internal consistency and test-retest reliability, criterion and construct validity.
**Clinic Staff Measures**		
Maslach Burnout Inventory, Human Services Survey for Medical Personnel© [50]	Professional burnout	50-item scale derived from the Human Services Survey for Medical Personnel assessing degree of emotional exhaustion, depersonalization, and personal accomplishment in work. This measure is validated by the extensive research that has been conducted for more than 35 years with this tool.
AAP Periodic Survey of Fellows (adapted) [51]	Screening and referral practices to address SDoH	This national survey has collected more than 75,000 pediatrician responses on hundreds of topics to inform policy, education, and advocacy. This measure is validated by the extensive research that has been conducted for more than 20 years with this tool.

Licenses were required and obtained for the following instruments: BITSEA; CD-RISC; Maslach Burnout Inventory Copyright©1981, 2016 by Christina Maslach & Susan E. Jackson; PICCOLO; PPAM; SEEK™© University of Maryland, Baltimore 2016

### Electronic Health records

The study used electronic health record (EHR) and Medicaid administrative data to examine trends in quality of care, health care utilization and financial impact of the innovations. Outcomes assessed include: health care quality (adherence to immunization and well-child visit schedules; developmental, maternal depression, and lead screening; and continuity of care); ambulatory care-sensitive hospitalizations; avoidable Emergency Department (ED) visits; and urgent care use among children served in the participating clinics. These data were used to analyze Medicaid spending on service use (recommended preventive versus urgent care/hospitalization) to inform the cost value of the innovations. We extracted the EHR and Medicaid data for all children age birth to two served in the participating study clinics from January 1, 2014 (corresponding with implementation of the Affordable Care Act) to February 29, 2020 (corresponding to study end). For each outcome using these data, analyses compare children’s health care and ED use before and after implementation of the innovations, as well as outcomes for children who did and did not receive the innovations.

## Results

The final sample recruited into the study included 908 parents, primarily mothers (97.6%) whose average age was 29.5 years. Nearly two-thirds reported being in two-parent households (married = 42.2%, domestic partner = 26.8%). Additionally, the sample was predominately comprised of parents who reported Hispanic ethnicity (69.2%). Children in the sample were 46.9% male. Finally, although the mean income across the sample of $37,788 depicted a moderate income-level, income varied substantially across the clinic sites. Of note, families at six of the clinics reported average income near or below the Federal Poverty Line for a family of 5 (FPL = $30,170). Range of annual family income was $0 to $550,000. The enrollment interview was completed by 98% of the recruited sample (*n* = 888); 764 parents completed the midpoint interview (84% of recruited sample), and 707 parents completed the final interview (78% of recruited sample). Table 3 presents sample demographics.

**Table 3:** Sample description

Caregiver Characteristics		
Caregiver relationship to child (*n* = 886)	**Frequency**	**%**
Mother	865	97.6
Father	12	1.4
Grandparent	3	0.3
Foster Parent	4	0.5
Legal Guardian	2	0.2
Marital Status (*n* = 882)		
Married	372	42.2
Domestic Partnership	236	26.8
Single	244	27.7
Separated	16	1.8
Divorced	12	1.4
Widowed	2	0.2
Ethnicity (*n* = 887)		
Hispanic	614	69.2
White (non-Hispanic)	125	14.1
Black (non-Hispanic)	106	12.0
Asian	15	1.7
American Indian or Alaskan Native	2	0.2
Middle Eastern or North African	3	0.3
Pacific Islander	3	0.3
More than one race	7	0.8
Other race	12	1.4
	**Mean (S.D.)**	**Range**
Caregiver age (*n* = 889)	29.5 (6.09)	18–55 years
**Child Characteristics**	**Mean (S.D.)**	**Range**
Child age (in months)	2.6 (1.7)	0.1–8 months
Child sex (*n* = 785)	**Frequency**	**%**
Female	417	53.1
Male	368	46.9
**Household Characteristics**	**Mean (S.D.)**	**Range**
Household size (*n* = 885)	5.2 (2.2)	2–20 people
Annual household income (*n* = 786)	$37,788 (44476)	$0 - $555,000
Annual household income by clinic		
Clinic A (*n* = 60)	$60,735 (41999)	$0 - $180,000
Clinic B (*n* = 120)	$29,263 (3698)	$1200 - $360,000
Clinic C (*n* = 117)	$30,991 (3467)	$9000 - $360,000
Clinic D (*n* = 118)	$22,293 (9434)	$0 - $52,000
Clinic E (*n* = 84)	$26,597 (32431)	$0 - $288,000
Clinic F (*n* = 112)	$27,340 (20375)	$1200 - $16,800
Clinic G (*n* = 101)	$78,797 (78108)	$5200 - $550,000
Clinic H (*n* = 21)	$20,517 (15787)	$5688 - $84,000
Clinic I (*n* = 53)	$4921 (46258)	$500 - $200,000

## Discussion

This paper describes the developmental approach used to execute a comprehensive, multi-level investigation of five communities and nine pediatric health care clinics implementing approaches to bolster screening and support for families of infants to address toxic stress and social determinants of health. We used this developmental evaluation approach to describe the early stages of adoption of the innovations and to tell the story of the intersecting systems essential to addressing the multi-factorial nature of toxic stress on families with young children. The layered community, pediatric clinic, and family perspectives form a comprehensive view of families with infants and their experiences with healthcare and community services. We used rapid-cycle feedback and co-interpretation approaches to maintain study engagement, and to elicit insight on preliminary findings from innovation developers, community providers, clinics, and families. This was a critical opportunity to gather early reactions from data sources to enhance our understanding of local context and add credibility of findings. This also ensured that clinics could respond to early findings to refine their service approaches.

The study answers questions about the value of social need screening and referral systems utilizing two approaches; that of having an embedded family specialist and legal consultation in medical practices (such as DULCE) and the other using existing health care practitioners and a multi-practice learning collaborative approach (such as I-SCRN). We do not compare the approaches’ outcomes given the developmental nature of their implementation, but rather we draw themes and implications that indicate the strengths and challenges to the implementation of each from multiple perspectives. We focus on barriers and facilitators to families connecting to and accepting services (such as early developmental interventions) as one of the significant challenges that must be addressed in the near term if the innovations are to be successful. Our integrated study of Help Me Grow in three communities aims to increase understanding of the role of a centralized referral system, such as HMG, in facilitating referrals that originate from the increased screening and family engagement approaches occurring in pediatric health care clinics. Primary care practices are stretched thin in their efforts to connect families to needed social services [52, 53].

The study advances policy and practices in the field of health care and community partnerships that will help to reduce the health implications of toxic stress exposure and lack of protective factors in infancy and early childhood. There is a business case that is central to integrating innovations such as those described in this study into pediatric health care: the potential for improved population health and reduced system cost over the life course, first among children—and later adolescents and adults—a preventive service model that is intended to shore up family access to resources early in children’s lives so that children do not experience chronic stress that can have health impact [54].

A strength of the study is that it was able to find common ground across five communities and nine clinics about these approaches to evaluation. An invaluable asset was being able to join an already established network, Early Childhood Learning and Innovation Network for Communities, where a commitment to data-driven decision-making and evidence creation was valued. While developmental evaluations of innovation adoption, implementation, and sustainability are often focused on single innovations [55], we believe there is value added in bringing the same questions and approaches to multiple approaches addressing social determinants of health. This paper is limited to describing the methodological approaches; outcomes are not addressed here but are being prepared for publication.

Health care practices considering screening and referral innovations for social needs in their medical setting face three primary challenges. First, because there is yet insufficient evidence that investing in innovations to address families’ social needs and reduce toxic stress will result in intended outcomes of improved health, increased health care quality, and cost savings, implementing these approaches currently requires practices to take a significant “leap of faith.” Health care systems and payors need the backing of rigorous research and evaluation to make a business case for the models and this is not yet possible. There is preliminary evidence of similar models in the area of increasing families’ access to resources and improving socio-economic circumstances. For example, using the WE CARE model to systematically screen and refer families to services, results of a randomized control trial including 336 mothers in 8 community health centers showed that mothers who received the intervention were more likely to be employed, less likely to be homeless, and to have accessed at least one resource compared with mothers who did not receive the intervention [56]. Studies such as this are promising, but more is needed to particularly understand health impact. More evidence is needed about what works best for whom and in what context, the key ingredients to successful approaches, and the approaches that show the most cost efficiency.

Second, health care in the U.S. does not yet have a universal administrative framework to pay providers to implement screening and the service coordination and referral roles that accompany identifying families’ social needs. A number of U.S. federal funding initiatives were launched in 2016–2017 to spark innovation through the Center for Medicare and Medicaid Innovation (CMMI). The initial phase evaluation results show challenges in areas such as having an adequate work force to address social needs, lack of standardization, adequate data systems, and closing referral loops between health care and community services [57, 58]. Medicaid demonstration programs are testing different payment and delivery models that include broadening services to health-related needs such as providing community health workers to assist families with housing, food and income [59, 60]. The recently introduced Social Determinants Accelerator Act of 2019 would convene a federal inter-agency technical advisory council to identify opportunities for state and local governments to coordinate funding and administration of federal programs that may be underutilized or unknown. These emerging approaches will provide many lessons and eventually provide direction for more widespread adoption if positive financial and quality of care impacts are realized.

Finally, health care practices need to consider the strengths and gaps in their partnerships with community service providers. Linkages between health care and community services are absent in most communities and building these systems takes time. Our study identifies social needs reported by families of infants receiving pediatric primary care. From family interview data, we will create profiles of families formed by common characteristics in the areas of risk and resilience and assess differences in health care engagement and longitudinal child and family outcomes. This will help health care stakeholders prioritize and tailor services to meet common social needs.

There are important limitations of this study. First, generalizability to under-served populations is limited. Black/African Americans, Asian, and indigenous people were less represented across the clinic sites and may have different experiences to share. Second, recruiting families to participate in focus groups that are held at a specific time/location was challenging. The intention was to have larger numbers participate but interest, feasibility with infants and young children, timing and transportation obstacles limited the size of some groups. Child care was provided during focus groups to facilitate participation. Third, clinic electronic health records (EHR) were maintained in highly disparate formats and accessibility, making it challenging to address questions across multiple states, communities, and clinics. While all of the clinics had EHR, the accessibility to data, functionality of the systems, documentation, and the ease of use created significant challenges. Finally, the study concluded when the children of the families were under the age of 2. Studying families beyond infancy will permit greater insights into the role that parent engagement with clinics and early childhood community services play in empowering families and whether their early experience with screening and referral services in their child’s infancy has an enduring impact on health and wellness.

## Conclusions

This study is one of the first comprehensive investigations of health innovations in primary care designed to prevent and mitigate toxic stress. Our articulated theory of change asserts that the effects of systems change, provider behavior, and family engagement efforts will continue to evolve and as a consequence of this change, families will experience increased and enhanced health and concrete support, in turn, affecting parent sense of agency and behavioral change that promotes child health and well-being. As the number of families living in stressful environmental conditions exacerbated by poverty continues to increase in the U.S., it is critical to develop effective methods to mitigate the impacts of toxic stress. Crucially needed are cost-effective supports for families that occur at critical transition points, are driven by families, and sustainable within the existing funding structures. The products of the evaluation will serve as benchmark evidence in the field and provide not only a vehicle for clarifying approaches to reducing stress but seed a basis for beginning to discern critical ingredients that inform replication. Investigating how innovations arise and impact families in pediatric primary care-community service partnerships that provide services to low-income as well as middle-income families allows the documentation of under-told stories of family resilience and strain to systems serving families.

## Data Availability

The datasets generated and/or analyzed during the current study are not publicly available but are available from the corresponding author on reasonable request.

## Abbreviations

ACEs: Adverse Childhood Experiences
AAP: American Academy of Pediatrics
BITSEA: Brief Infant-Toddler Social and Emotional Assessment
CD-RISC: Connor-Davidson Resilience Scale
CIRP: Clinic Inclusivity and Regard for Parents Scale
CSSP: Center for the Study of Social Policy
CMMI: Center for Medicare and Medicaid Innovation
DULCE: Developmental Understanding and Legal Collaboration for Everyone
EC-LINC: Early Childhood Learning and Innovation Network for Communities
ED: Emergency Department
EHR: Electronic Health Records
FITS-P: Functional Impact of Life Events for Parents
FPL: Federal Poverty Line
HMG: Help Me Grow
IDQ: Infant Development Questionnaire
IRB: Institutional Review Board
I-SCRN: Improving Screening, Connections with Families, and Referral Networks
NSCH: National Survey of Children’s Health
PPAM: Parent Patient Activation Measure
PICCOLO: Parenting Interactions with Children: Checklist of Observations Linked to Outcomes
PSS: Parental Stress Scale
SEEK™: Safe Environment for Every Kid
SDoH: Social determinants of health
U.S.: United States
U.S. DHHS: U.S. Department of Health and Human Services

## References

[1] World Health Organization, Commission on Social Determinants of Health (2008). Closing the Gap in a Generation: Health Equity through Action on the Social Determinants of Health. Final Report of the Commission on Social Determinants of Health.

[2] World Health Organization (2020). Social Determinants of Health.

[3] Dankwa-Mullan I, Rhee KB, Stoff DM, Pohlhaus JR, Sy FS, Stinson N, Ruffin J (2010). Moving toward paradigm-shifting research in health disparities through translational, transformational, and transdisciplinary approaches. Am J Public Health.

[4] Rosen Valverde JN, Backstrand J, Hills L, Tanuos H (2018). Medical-legal partnership impact on parents’ perceived stress: a pilot study. Behav Med.

[5] Sandel M, Sheward R, Ettinger de Cuba S, Coleman S, Heeren T, Black MM, Casey PH, Chilton M, Cook J, Cutts DB, Rose-Jacobs R, Frank DA (2018). Timing and duration of pre- and postnatal homelessness and health of young children. Pediatrics.

[6] Brisson D, McCune S, Wilson JH, Speer SR, McCrae JS, Calhoun KH (2020). A systematic review of the association between toxic stress and poverty. J Evid Base Soc Work.

[7] Roggman LA, Roggman L, Cardia N (2016). Home visiting to promote developmental parenting: measurement to ensure quality. Home visitation programs: preventing violence and promoting healthy early childhood.

[8] Shonkoff JP, Garner AS, The Committee on Psychosocial Aspects of Child and Family Health, Committee on Early Childhood, Adoption, and Dependent Care, Section on Developmental and Behavioral Pediatrics (2012). The lifelong effects of early childhood adversity and toxic stress. Pediatrics.

[9] Bethell C, Jones J, Gombojav N, Linkenback J, Sege R (2019). Positive childhood experiences and adult mental and relational health in a statewide sample. JAMA Pediatr.

[10] Daniel H, Bornstein SS, Kane GC (2018). Addressing social determinants to improve patient care and promote health equity: an American College of Physicians position paper. Ann Intern Med.

[11] Garner AS, Shonkoff JP, Siegel BS, Dobbins MI, Earls MF, Garner AS, Wood DL (2012). Early childhood adversity, toxic stress, and the role of the pediatrician: translating developmental science into lifelong health. Pediatrics.

[12] King TM, Tandon D, Macias MM, Healy JA, Duncan PM, Swigonski NL, Skipper SM, Lipkin PH (2010). Implementing developmental screening and referrals: lessons learned from a national project. Pediatrics.

[13] Radecki L, Sand-Loud N, O’Connor KG, Sharp S, Olson LM (2011). Trends in the use of standardized tools for developmental screening in early childhood: 2002–2009. Pediatrics.

[14] Adams EK, Johnston EM, Guy G, Joski P, Ketsche P (2019). Children’s health insurance program expansions: what works for families?. Glob Pediatric Health.

[15] U.S. Dept. of Health and Human Services, Office of Minority Health (2017). Minority Population Profiles.

[16] Berkowitz SA, Basu S, Gundersen C, Seligman HK (2019). State-level and county-level estimates of health care costs associated with food insecurity. Prev Chronic Dis.

[17] Carroll A, Corman H, Curtis MA, Noonan K, Reichman NE (2017). Housing instability and children’s health insurance gaps. Acad Pediatr.

[18] Urban Institute. With public charge rule looming, one in seven adults in immigrant families reported avoiding public benefit programs in 2018: Urban Institute; 2018. https://www.urban.org/urban-wire/public-charge-rule-looming-one-seven-adults-immigrant-families-reported-avoiding-public-benefit-programs-2018. Accessed 12 Jan 2020.

[19] Sege R, Preer G, Morton SJ, Cabral H, Morakinyo G, Lee V, Abreu C, De Vos E, Kaplan-Sanoff M. Medical-legal strategies to improve infant health care: A randomized trial. Pediatrics. 2015;136(1). 10.1542/peds.2014-2955. 10.1542/peds.2014-2955 PMC9923600 26034248

[20] Flower K, Earls M, Nagy B, Janies K, Massie S, Bassewitz J. I-SCRN: A quality improvement collaborative to increase early childhood screening, referral, and follow-up in pediatric primary care practices. Pediatrics. 2019;144(2). 10.1542/peds.144.2_MeetingAbstract.72.

[21] Flower KB, Massie S, Janies K, Bassewitz JB, Coker TR, Gillespie RJ, Macias MM, Whitaker TM, Zubler J, Steinberg D, DeStigter L, Earls MF. Increasing early childhood screening in primary care through a quality improvement collaborative. Pediatrics. 2020;146(3). 10.1542/peds.2019-2328. 10.1542/peds.2019-2328 32769199

[22] Help Me Grow. The Help Me Grow System Model: Help Me Grow National Center; 2020. https://helpmegrownational.org/hmg-system-model/. Accessed 29 June 2020.

[23] Gottfriedson DC, Cook TD, Gardner FEM, Gorman-Smith D, Howe GW, Sandler IN, Zafft KM (2015). Standards of evidence for efficacy, effectiveness, and scale-up research in prevention science: next generation. Prev Sci.

[24] Beckett K, Farr M, Kothari A, Wye L, le May A (2018). Embracing complexity and uncertainty to create impact: exploring the processes and transformative potential of co-produced research through development of a social impact model. Health Res Policy Sys.

[25] Patton MQ (2010). Developmental evaluation: applying complexity concepts to enhance innovation and use.

[26] Birt L, Scott S, Cavers D, Campbell C, Walter F (2016). Member checking: a tool to enhance trustworthiness or merely a nod to validation?. Qual Health Res.

[27] Dubowitz H, Feigelman S, Lane W, Kim J (2009). Pediatric primary care to help prevent child maltreatment: the safe environment for every kid (SEEK) model. Pediatrics.

[28] Dubowitz H, Feigelman S, Lane W, Prescott L, Blackman K, Grube L, Meyer W, Tracy JK (2007). Screening for depression in an urban pediatric primary care clinic. Pediatrics.

[29] Dubowitz H, Prescott L, Feigelman S, Lane W, Kim J (2008). Screening for intimate partner violence in a pediatric primary care clinic. Pediatrics.

[30] Lane W, Dubowitz H, Feigelman S, Prescott L, Meyer W, Tracy JK (2007). Screening for parental substance abuse in pediatric primary care. Ambul Pediatr.

[31] Fauth RC, Leventhal T, Brooks-Gunn J (2004). Short-term effects of moving from public housing in poor to middle-class neighborhoods on low-income, minority adults’ outcomes. Soc Sci Med.

[32] Rollins C, Glass NE, Perrin NA, Billhardt KA, Clough A, Barnes J, Hanson GC, Bloom TL (2012). Housing instability is a strong a predictor of poor health outcomes as level of danger in an abusive relationship: findings from the SHARE study. J Interpers Violence.

[33] Burke Harris N, Renschler T (2015). Center for Youth Wellness ACE-questionnaire (CYW ACE-Q child, teen, teen SR).

[34] Moreno A (2016). Functional impact of life events for parents (FITS-P). Unpublished measure.

[35] Connor KM, Davidson JR (2003). Development of a new resilience scale: the Connor Davidson resilience scale (CD-RISC). Depress Anxiety.

[36] Pearlin LI, Schooler C (1978). The structure of coping. J Health Soc Behav.

[37] Pearlin LI, Lieberman M, Menaghan E, Mullan J (1981). The stress process. J Health Soc Behav.

[38] Turner RJ, Noh S (1988). Physical disability and depression: a longitudinal analysis. J Health Soc Behav.

[39] Rahman A, Iqbal Z, Robert C, Husain N (2009). Cluster randomized trial of a parent-based intervention to support early development of children in a low-income country. Child Care Health Dev.

[40] Berry JO, Jones WH (1995). The parental stress scale: initial psychometric evidence. J Soc Pers Relat.

[41] Norman V, Christiansen K (2013). Validity of the PICCOLO tool in child care settings: can it assess caregiver interaction behaviors?. Infant Ment Health J.

[42] Briggs-Gowan MJ, Carter AS, Irwin JR, Wachtel K, Cicchetti DV (2004). The Brief Infant-Toddler Social and Emotional Assessment: Screening for social-emotional problems and delays in competence. J Pediatr Psychol.

[43] Briggs-Gowan MJ, Carter AS, McCarthy K, Augustyn M, Caronna E, Clark R (2013). Clinical validity of a brief measure of early childhood social-emotional/behavioral problems. J Pediatr Psychol.

[44] Mathematica Policy Research, Inc. & the Administration on Children, Youth, and Families, U.S (1998). Department of Health and Human Services. Early Head Start Parent Interview: For Parents of 14-Month-Old Infants.

[45] LeCroy C, Milligan Associates, Inc (2004). Healthy Families Parenting Inventory. Unpublished users Manual.

[46] Krysik J, LeCroy CW. Development and initial validation of an outcome measure for home visitation: the Healthy Families Parenting Inventory. Infant Ment Health J. 2012;33(5):496–505. 10.1002/imhj.21343. 10.1002/imhj.21343 28520271

[47] Help Me Grow (2020). The Help Me Grow System Model. Help Me Grow National Center.

[48] Insignia Health (2011). Parent Patient Activation Measure.

[49] Hibbard JH, Stockard J, Mahoney ER, Tusler M (2004). Development of the patient activation measure (PAM): conceptualizing and measuring activation in patients and consumers. Health Serv Res.

[50] Maslach C, Jackson S, Leiter M. Maslach Burnout Inventory: manual and non-reproducible instrument and scoring guides; forms included: general (MBI – GS), human services (MBI – HSS), et educators (MBI – ES). Menlo Park: Mind Garden; 2013.

[51] American Academy of Pediatrics Periodic Survey of Fellows. Menlo Park: 2017. https://www.aap.org/en-us/professional-resources/Research/pediatrician-surveys/Pages/survey-findings.aspx. Accessed 22 Mar 2017.

[52] LaForge K, Gold R, Cottrell E, Bunce AE, Proser M, Hollombe C, Clark KD (2018). How 6 organizations developed tools and processes for social determinants of health screening in primary care. J Ambul Care Manage.

[53] Berry C, Paul M, Massar R, Marcello RK, Krauskopf M (2020). Social needs screening and referral program at a large US public hospital system, 2017. Am J Public Health.

[54] Murray G, Rodriguez H, Lewis V (2020). Upstream with a small paddle: how ACOs are working against the current to address patients’ social needs. Health Aff.

[55] Miller TR, Hendrie D (2015). Nurse family partnership: comparing costs per family in randomized trials versus scale-up. J Prim Prev.

[56] Garg A, Toy S, Tripodis Y, Silverstein M, Freeman E (2015). Addressing social determinants of health at well child care visits: a cluster RCT. Pediatrics.

[57] Beil H, Feinberg RK, Patel SV, Romaire MA (2019). Behavioral health integration with primary care: implementation experience and impacts from the state innovation model round 1 states. Milbank Q.

[58] Tumber MB, Bunzli L, Rosenberg M (2019). Addressing the social determinants of health: the Rhode Island state innovation model (RI SIM) experience. Rhode Island Med J.

[59] Alley DE, Asomugha CN, Conway PH, Sanghavi DM (2016). Accountable health communities – addressing social needs through Medicare and Medicaid. N Engl J Med.

[60] Hinton E, Artiga S, Musumeci M, Rudowitz R (2019). A first look at North Carolina’s section 1115 Medicaid waiver’s healthy opportunities pilots.

